# Current and Emerging MR Methods and Outcome in Rodent Models of Parkinson’s Disease: A Review

**DOI:** 10.3389/fnins.2021.583678

**Published:** 2021-04-07

**Authors:** Alexandra Petiet

**Affiliations:** ^1^Centre de Neuroimagerie de Recherche, Institut du Cerveau, Paris, France; ^2^Inserm U1127, CNRS UMR 7225, Sorbonne Universités, Paris, France

**Keywords:** MRI methods, Parkinson’s disease, animal model, diffusion MRI, MR spectroscopy, resting-state functional MRI

## Abstract

Parkinson’s disease (PD) is a major neurodegenerative disease characterized by massive degeneration of the dopaminergic neurons in the substantia nigra pars compacta, α-synuclein-containing Lewy bodies, and neuroinflammation. Magnetic resonance (MR) imaging plays a crucial role in the diagnosis and monitoring of disease progression and treatment. A variety of MR methods are available to characterize neurodegeneration and other disease features such as iron accumulation and metabolic changes in animal models of PD. This review aims at giving an overview of how those physiopathological features of PD have been investigated using various MR methods in rodent models. Toxin-based and genetic-based models of PD are first described. MR methods for neurodegeneration evaluation, iron load, and metabolism alterations are then detailed, and the main findings are provided in those models. Ultimately, future directions are suggested for neuroinflammation and neuromelanin evaluations in new animal models.

## Introduction

Parkinson’s disease is a major neurodegenerative disease in the elderly affecting 7 to 10 million people worldwide. This disease is characterized by massive degeneration of the dopaminergic (DA) neurons and Lewy body inclusions containing α-synuclein proteins in the subtantia nigra pars compacta (SNc), as well as neuroinflammation. The reduction of DA levels in the striatum (STR) causes the appearance of the clinical symptoms such as akinesia, rigidity, and tremor. The clinical diagnosis can only be done when 50% of those neurons are destroyed (Redgrave et al., 2010). While most forms of PD are sporadic, less than 10% are associated with familial mutations (Dauer and Przedborski, 2003; Thomas and Beal, 2007). Mutations in the *leucin-rich repeat kinase 2* (*LRRK2*) and the α*-synuclein coding* gene (*SNCA*) are responsible for the autosomal-dominant PD, and mutations in the *Parkin* (*PARK2*), the *phosphatase and tensin (Pten)-induced kinase 1* (*PINK1*), and *DJ-1* genes are responsible for the autosomal–recessive PD.

**FIGURE 1:**
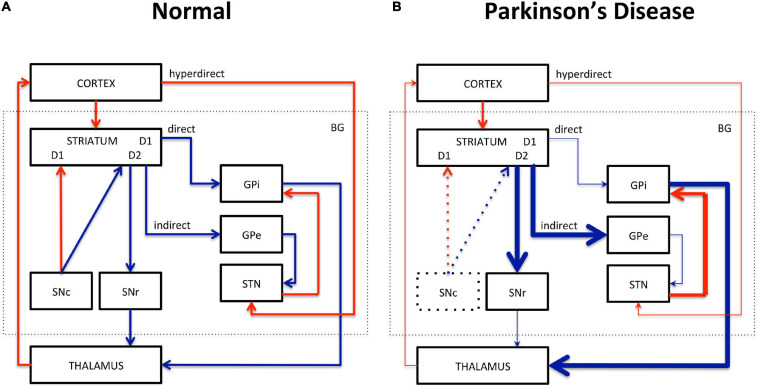
Schematic representation of the corticobasal ganglia–thalamocortical motor pathway model. **(A)** Normal pathway. **(B)** PD pathway. Red arrows correspond to glutamatergic excitatory projection neurons. Blue arrows correspond to GABAergic inhibitory projection neurons. D_1_, D_2_: striatal receptors. SNc, subtantia nigra pars compacta; SNr, subtantia nigra pars reticulata; STN, subthalamic nucleus; GPi, globus pallidus interna; GPe, globus pallidus externa; BG, basal ganglia. The direct (STR/GPi/TH), indirect (STR/GPe/STN/GPi/TH), and hyperdirect (cortex/STN) pathways are represented. Thick lines represent increased excitation/inhibition. Thin lines represent decreased excitation/inhibition. Dotted lines represent DA neuronal loss in the SNc.

Motor dysfunction in PD is classically described by the corticobasal ganglia–thalamocortical motor pathway model and with the direct, indirect, and hyperdirect pathways illustrated in Figure 1 (Delong, 1990; Honey et al., 2003; Lanciego et al., 2012). The basal ganglia (BG) include the dorsal STR, the globus pallidus (GP) interna and externa, the SNc, the substantia nigra pars reticulata (SNr), the thalamus (TH), and the subthalamic nuclei (STN). The dorsal STR receives excitatory glutamatergic (Glu) inputs from the cortex, while projection neurons from the SN and GP to the TH use inhibitory γ-aminobutyric acid (GABA) neurotransmitters. To close the loop, the motor cortex receives back excitatory Glu projections from the TH. Consequently, inhibition of the TH therefore leads to inhibition of the motor activation. Within the BG, the direct pathway is composed of monosynaptic connections from the dorsal STR to the GP interna, whereas the indirect pathway is composed of polysynaptic connections from the dorsal STR to the GP externa, STN, and to the GP interna. The hyperdirect pathway projects cortical neurons directly to the STN. Coming from the SNc, DA excites or inhibits GABAergic medium spiny neurons via D_1_ or D_2_ receptors in the STR, respectively. Those structures and neurotransmitters have been the targets for neuroimaging methods developments.

Neuroinflammation also characterizes PD and includes microglial activation, astrocyte proliferation, lymphocyte infiltration, and the presence of proinflammatory cytokines (Glass et al., 2010). Whether inflammation is a cause or consequence of neurodegeneration in PD is still under debate (de Lau and Breteler, 2006; Glass et al., 2010; Tansey and Goldberg, 2010; Cabezudo et al., 2020). Likewise, the protective or deleterious role of inflammation in PD is still unknown (Sofroniew, 2015).

Non-invasive imaging tools have the potential to add critical value for earlier diagnosis and therefore for the development of more efficient treatments against the disease. In this regard, Magnetic Resonance Imaging (MRI) plays a crucial role in the diagnosis and monitoring of disease progression and treatment in both humans and animal models. Neurodegeneration has been evaluated in the SN and nigrostriatal fibers in PD patients using MRI. Changes in diffusion metrics have been reported in the SN of patients [decreased anisotropy and increased mean diffusivity (MD)] and spreading to other gray and white matter regions and tracts, indicating expended loss of microstructural integrity (see review by Weingarten et al., 2015). Likewise, changes in the functional properties of these structures can be detected with resting-state functional MRI (rs-fMRI) (see review by Lehéricy et al., 2012). Reduced functional connectivity (FC) in the SN and in corticostriatal networks was also reported in patients (Helmich et al., 2010; Sharman et al., 2012).

Abnormal iron accumulation in specific brain regions has been observed in several neurodegenerative diseases including PD. Up to 35% increase in the SNc of PD patients has been reported (Dexter et al., 1989; Hirsch et al., 1991). As T_2_- and T_2_^∗^-weighted imaging are sensitive to the presence of paramagnetic iron, they have been used to evaluate iron deposits in the SN (Lehéricy et al., 2014; see review by Pietracupa et al., 2017). Decreased T_2_ values have been measured in the caudate nucleus, putamen, and SN of PD patients (Antonini et al., 1993). Increased nigral transverse relaxation rates *R*_2_^∗^ (1/T_2_^∗^) have been reported in PD patients and correlated with disease progression (Ulla et al., 2013; Hopes et al., 2016).

Metabolism alterations in PD patients have been investigated with MR spectroscopy (MRS). While N-acetyl-aspartate (NAA) is a marker of neuronal integrity, myo-inositol (mIns) is a marker of gliosis, and creatine (Cre) is a marker of energy metabolism. Changes in neurotransmitters levels can also inform on the pathological state. For instance, decreased levels of NAA have been reported in the cortex and SN, whereas increased GABA and Glu levels have been found in the pons, putamen, and SN of PD patients (Emir et al., 2012; Levin et al., 2012; Graff-Radford et al., 2014; Gröger et al., 2014).

Animal models have been widely used to improve our understanding of PD features with anatomical, functional, and metabolic MR-based tools. Toxic models based on intracerebral or systemic injections of neurotoxins produce nigrostriatal lesions that replicate many of PD features (see the review by Blandini and Armentero, 2012). The most used models of neurodegeneration are based on injections of the 6-hydroxydopamine (6-OHDA) and the 1-methyl-4-phenyl-1,2,3,6-tetrahydropyridine (MPTP) neurotoxins to produce degeneration of DA neurons in the SN and subsequently of the entire nigrostriatal pathway (Yuan et al., 2005). Although those models are partial models and more acute than the progressive human pathology, they have been extremely useful in mimicking many of PD features accompanied by motor symptoms. Alternatively, genetic-based animal models of PD allow the investigation of the prodromal stage of the disease during the presymptomatic period, as well as the study of specific pathways related to genetic and biochemical alterations (see reviews by Chesselet et al., 2008; Creed and Goldberg, 2018 and by Dawson et al., 2010).

This review aims at giving an overview of how the physiopathological features of PD have been investigated using the most represented MR methods in animal models. It is based on PubMed searches within the last 10 years, and it is limited to rodent models of PD as they have been the most widely used and for their potential in genetic studies. It was elaborated based on a physiopathological perspective to demonstrate how various imaging approaches have been used to investigate PD physiopathology in animals. Rodent models are first described, including toxin-based and genetic-based models. Then structural and functional MR methods are detailed, such as diffusion and rs-fMRI to evaluate neurodegeneration, followed by T_2_^∗^ and susceptibility imaging to evaluate iron accumulation and MRS to evaluate metabolism changes. Future directions for preclinical MR developments are suggested and include strategies for neuroinflammation and neuromelanin evaluations.

## Rodent Models

### Toxin-Based Models

#### The 6-OHDA Model

Following the discovery that 6-OHDA could produce selective degeneration of sympathetic adrenergic nerves (Thoenen and Tranzer, 1968), this neurotoxin has been used as a denervation tool in animals (Ungerstedt, 1968; Jonsson, 1980). As a hydroxylated analog of DA, 6-OHDA enters DA neurons through DA transporters. Once in the cytosol, it forms hydrogen peroxide by auto-oxidation reaction. As 6-OHDA does not cross the blood-brain barrier (BBB), it is necessary to administer it directly into the brain with stereotaxic injections. As bilateral injections cause high mortality rates, unilateral injections have been preferred. The mechanism of action varies depending on the injection site along the nigrostriatal pathway. Injections into the SNc or into the medial forebrain bundle (MFB) produce massive and rapid anterograde degeneration of the nigral DA neurons, up to 90%–100% of SN and striatal neurons, and subsequently of the entire nigrostriatal pathway within days. Alternatively, injections into the dorsal STR induce partial lesions of the nigral DA neurons, up to 50%–70% loss within 4 to 6 weeks, which leads to progressive retrograde degeneration of the nigrostriatal pathway more closely mimicking human pathology and allowing longitudinal evaluations (Berger et al., 1991; Przedborski et al., 1995; Shimohama et al., 2003). In the STR, greater than 90% loss of DA can be reached after intrastriatal infusion of 6-OHDA in mice (Xiao et al., 2011).

#### The MPTP Model

The selective toxicity of MPTP for the nigrostriatal tract was first described by Langston et al. (1983). MPTP is converted to 1-methyl-4-phenylpyridinium ion (MPP^+^) and accumulates in the SNc neurons via DA transporters. As MPTP can cross the BBB, it can be injected via the peripheral system. However, systemic MPTP administration fails in rats as the conversion from MPTP to MPP^+^ occurs at the BBB preventing influx into the brain (Giovanni et al., 1994; Pienaar et al., 2010; Jagmag et al., 2016; Konnova and Swanberg, 2018); it is therefore alternatively used in mice (Mori et al., 1988). Repeated intraperitoneal injections in mice cause rapid and massive DA neuron loss, which leads to similar symptoms as those found in patients such as akinesia, rigidity, and episodes of tremor (Jackson-Lewis et al., 1995; Tatton and Kish, 1997).

### Genetic-Based Models

#### Autosomal-Dominant Models

The most common mutations in autosomal-dominant PD are the *LRRK2* mutations (Zimprich et al., 2004). This kinase enzyme is normally present in membranes and plays a role in mitochondria, autophagy, and endocytosis (Winklhofer and Haass, 2010; Berwick et al., 2019). Transgenic mice present little to no DA neurodegeneration; however, most of them have abnormalities in the nigrostriatal system, α-synuclein aggregation, or impaired DA release (Li et al., 2009; Jagmag et al., 2016). Likewise, *LRRK2* mutated rats do not show any DA neurodegeneration in the SN but rather behavioral alterations (Daher et al., 2014; Walker et al., 2014; Lee et al., 2015; Shaikh et al., 2015; Sloan et al., 2016).

The *SNCA* gene codes for the presynaptic α-synuclein protein, which is abundantly found in the brain (Maroteaux et al., 1988). Its function is not fully understood; however, it is believed to play a role in synaptic vesicle function, hence of neurotransmitter release (Kahle et al., 2002). Overexpression of α-synuclein produces heterogeneous phenotypes in mice, depending on the promoters used for transgene expression. Although they lack DA degeneration, some of them present nigrostriatal dysfunctions (Abeliovich et al., 2000; Fleming et al., 2005; Chesselet et al., 2008).

#### Autosomal–Recessive Models

The *Parkin* (*PARK2*) gene is involved in the ubiquitin proteasome system as an E3 ubiquitin ligase, and mutations of this gene cause loss of function in patients. *Parkin* knockout does not seem to induce nigrostriatal or DA lesions in mice (Perez and Palmiter, 2005). However, overexpression of a mutated form of this gene leads to nigral DA cell depletion, striatal synaptic loss, and decreased striatal DA levels in mice (Lu et al., 2009). Likewise, overexpression of *Parkin* induces mild neurodegeneration in rats (Van Rompuy et al., 2014).

*PINK1* is a mitochondrial protein kinase that protects neurons from mitochondrial dysfunction stress. The *PINK1* mutation leads to loss of function mainly affecting the kinase domain in patients (Klein and Westenberger, 2012). *PINK1* knockout does not produce any DA neuronal depletion in mice, but it alters DA neurotransmission and mitochondrial function (Kitada et al., 2007; Gautier et al., 2008; Gispert et al., 2009). In contrast, *PINK1* knockout rats have SN DA neuronal loss, α-synuclein accumulation, mitochondrial defects, and motor dysfunction (Dave et al., 2014; Villeneuve et al., 2016).

*DJ-1* is a ThiJ/Pgpl molecular chaperone encoded by the *PARK7* gene and widely expressed in the body. *DJ-1* knockout mice do not exhibit any DA depletion but show some nigrostriatal and mitochondrial abnormalities (Goldberg et al., 2005; Kim et al., 2005; Andres-Mateos et al., 2007).

#### Other Models

A conditional knockout mouse model with respiratory chain-deficient DA neurons was created in 2007 and named MitoPark mice (Ekstrand et al., 2007). This model is based on the inactivation of the mitochondrial transcription factor A (*Tfam*) gene in DA neurons. Among the different genetic models, those mice present the most PD-like phenotypes including DA cell death, intraneuronal inclusions, and progressive motor dysfunction.

## MR Methods for Neurodegeneration Evaluation

### Diffusion Imaging

In an unrestricted medium, water molecules undergo random Brownian motion and diffuse freely. Its motion can be hindered by membranes, extracellular hindrance, or tissue heterogeneity. Diffusion-weighted imaging is sensitive to water diffusion through the application of diffusion gradients (Le Bihan, 2003). In the white matter, where fiber bundles constitute physical constraints, water molecules diffuse along a preferred direction along the fibers, which is referred to as anisotropy. The diffusion tensor model, a model of the displacement of water molecules, can provide indices such as the MD, characterizing the overall displacement of water molecules; the fractional anisotropy (FA), characterizing the orientation of diffusion; and the eigenvalues, characterizing the main directions of diffusivities, also derived as axial and radial diffusivities (AD, RD). Although the cellular origin of anisotropy is multifactorial and remains unclear (Chabert and Scifo, 2007), it has been shown that AD changes can be used as an index of axonal damage, whereas RD can be used as an index of myelin damage (Song et al., 2005). In highly oriented fiber bundles, FA is high (close to 1), whereas in regions of crossing fibers, it is low (close to 0).

**FIGURE 2:**
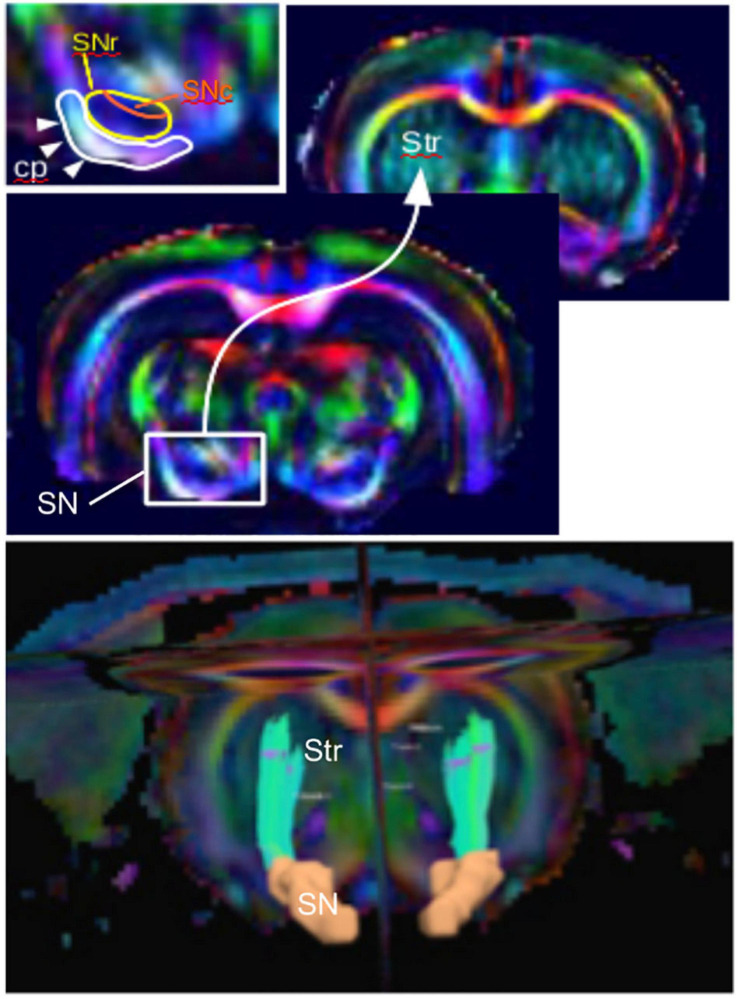
The nigrostriatal pathway illustrated with color-coded diffusion tensor images of a rat brain (coronal views). **(Top)** Zoom into the subtantia nigra (SN) projection to the striatum (Str) and inlet showing the detailed structure with the cerebral peduncle (cp), the SN reticulata (SNr), and the SN compacta (SNc). **(Bottom)** Three-dimensional reconstructed nigrostriatal tract (green) from the SN (orange) to the dorsal Str using diffusion-based tractography.

Diffusion imaging has been used to evaluate microstructural changes in rodents. The SN can be sufficiently resolved from those images, and the nigrostriatal tract can clearly be identified and reconstructed (Figure 2). Following MFB injections of 6-OHDA in rats, Monnot et al. (2017) reported decreased FA and increased RD in the ipsilateral SNc and SNr. Those results were supported by a previous study by Soria et al. (2011), which showed bilateral changes in the SN in the same model targeting the MFB. They showed that FA was decreased in the ipsilateral SNr, AD was bilaterally decreased in the SNr, and RD was bilaterally increased in the cortex (Soria et al., 2011). Furthermore, those results were consistent with neurodegeneration and with human findings (Vaillancourt et al., 2009; Rolheiser et al., 2011; Skorpil et al., 2012; Cochrane and Ebmeier, 2013; Schwarz et al., 2013). In contrast, they were in disagreement with a previous study by Van Camp et al. (2009), which found increased FA in the ipsilateral SN of 6-OHDA rats injected in the STR. This FA increase was attributed to neuroinflammation, but the discrepancy with the other studies might also be due to differences in the injection site (MFB vs. STR). A more recent study by Perlbarg et al. (2018) demonstrated increased FA in the ipsilateral and contralateral STR of 6-OHDA rats who received intrastriatal injections (Perlbarg et al., 2018). This result was consistent with neurodegeneration in a crossing-fiber structure. Indeed, regions of crossing fibers such as the STR have lower FA than regions of linearly oriented fibers such as corpus callosum (CC). Therefore, the selective degeneration of a group of fibers crossing other populations of fibers leads to increase of FA (Winston, 2012). Additionally, they found increased MD in the ipsilateral STR indicating loss of microstructural integrity and in line with human findings. However, in this study, no changes in the SN could be detected presumably because of a lack of sensitivity.

In genetic-based models, neuroimaging studies showed decreased MD, AD, and RD in regions of known α-synuclein accumulation such as the SN, STR, sensorimotor cortex (SM), and TH in α-synuclein transgenic mice (Supplementary data, Khairnar et al., 2015). Those results could be explained by the presumable decrease of free water diffusion caused by the protein aggregation. In a presymptomatic *PINK1* knockout rat model, altered diffusion metrics [reduced anisotropy and apparent diffusion coefficient (ADC)] were observed in the BG and other regions (e.g., hippocampus, brainstem, and cerebellum) (Cai et al., 2019). Those widespread changes throughout the brain have also been described in PD patients (Braak et al., 2004). In the MitoPark mouse model, decreased FA has been measured in the SN and CC, indicating neuronal and fiber degeneration (Cong et al., 2016).

### Functional Imaging

Functional connectivity or “synchrony” between and within brain regions refers to temporal correlations between spatially remote neurophysiological events, as measured by fMRI blood oxygenation level-dependent (BOLD) signal (Friston et al., 1993). Using fMRI, low-frequency, spontaneous, and, in some cases, coherent signal fluctuations may be detected in the resting brain (Biswal et al., 1995). Rs-fMRI studies have thus revealed co-activation in distributed networks of cortical and subcortical regions that characterize functional brain networks. Such connectivity may or may not also involve a structural connection (Honey et al., 2009). Most of PD imaging in patients and in animal models has been conducted in the resting state.

Functional connectivity changes have been explored in the nigrostriatal pathway in animal models. Decreased FC was found in the interhemispheric STR and in the ipsilateral cortices of 6-OHDA rats (Monnot et al., 2017; Westphal et al., 2017). Those animals were injected in the MFB and anesthetized with either a mixture of isoflurane and medetomidine (former study) or with medetomidine alone (latter study) during imaging. Likewise, decreased FC between the ipsilateral primary motor cortex (M1) and contralateral TH was reported in the intrastriatal 6-OHDA model using isoflurane alone (Perlbarg et al., 2018). Zhurakovskaya et al. (2019) also reported decreased FC in corticocortical and striatocortical connections of 6-OHDA-injected rats under urethane anesthesia (Zhurakovskaya et al., 2019). Decreased FC is commonly interpreted as direct lesioning effects.

**FIGURE 3:**
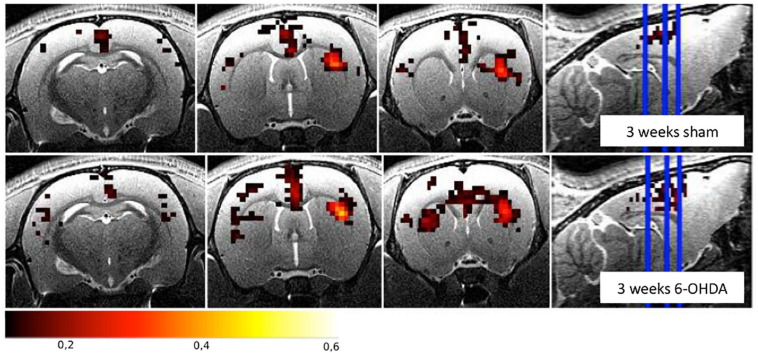
Functional connectivity maps from the right striatum for the sham-operated and the 6-OHDA groups at 3 weeks postinjection in the right hemisphere (i.e., on the right side of the brain on the images). The 6-OHDA group shows a non-significant increase of FC between the left and right striatum. Maps are expressed as correlation coefficient *R*. See Perlbarg et al. (2018) for the full study description.

Increased FC was found between the STR and the SM and in the TH of both hemispheres in 6-OHDA rats injected in the MFB (Monnot et al., 2017; Westphal et al., 2017). Similarly, increased FC was found between the ipsilateral STR and the GP, the contralateral M1 and the GP, and the interhemispheric STR and the GP of 6-OHDA rats injected in the STR (Perlbarg et al., 2018). Figure 3 illustrates the FC maps from the lesioned STR to the rest of the brain in this study and highlights the (non-significant) increased FC between the ipsilateral and contralateral STR in the 6-OHDA group compared to the sham-operated group 3 weeks after lesioning. Increased FC is generally attributed to compensatory effects and reorganization, like it has been observed in PD patients (Helmich et al., 2010; Sharman et al., 2012).

In a presymptomatic *PINK1* knockout rat model, Cai et al. (2019) observed changes in rs-fMRI connectivity in the BG and other regions such as the amygdala, cortex, septum, and pons. They measured decreased connectivity between the TH and STR, whereas the cerebellar nuclei showed increased connectivity within the cerebellum and hippocampus in *PINK1* rats under isoflurane anesthesia. Based on their findings, they suggested a reorganization of connectivity pathways in PINK1 mice, in which the STR to TH connection would be rerouted from the STR to the hippocampus also showing increased connectivity from the cerebellum. They argued on the role of the cerebellum in PD pathology similar to the cerebellar hyperconnectivity found in PD patients (Cerasa et al., 2016; Tuovinen et al., 2018).

## MR Methods for Iron Accumulation Evaluation

### T_2_^∗^ Imaging

It has been shown that iron accumulates in the SN of MPTP and 6-OHDA-lesioned animals (Wang et al., 2004; Hare et al., 2009; Jiang et al., 2010; Lv et al., 2011). Iron deposits can be detected with conventional T_2_^∗^ imaging. For example, Olmedo et al. (2017) quantified hyposignal levels in the SN of 6-OHDA rats 1 and 4 weeks postinjection in the MFB. They reported significantly increased hypointense pixels (i.e., decreased T_2_^∗^ signal) in 6-OHDA rats compared to sham rats, which correlated to iron staining with Prussian blue at 4 weeks. Furthermore, Virel et al. (2014) evidenced iron accumulation in the STR of 6-OHDA rats following intrastriatal injections. In this study, they measured increased T_2_^∗^ hypointensities (i.e., decreased T_2_^∗^ signal) in the ipsilateral STR as early as 1 week postlesioning and persisting up to 4 weeks. They also showed correlations of those hypointensities with edematous hyperintensities and with iron accumulation revealed with Prussian blue staining at 4 weeks only. In contrast, the SN remained intact, presumably due to milder and delayed depletion in this structure at this later timepoint.

Decreased T_2_^∗^ in the SN and STR has also been found in the MitoPark mouse model and was presumably attributed to iron accumulation in the SN as it is a shared feature with PD patients.

### Susceptibility Imaging

Other iron-sensitive MRI methods include susceptibility-weighted imaging (SWI) and quantitative susceptibility mapping (QSM), both of which have been used to improve imaging of the SN nigrosomes, STN, and GP interna (Lotfipour et al., 2012; Liu et al., 2013; Schwarz et al., 2014). In SWI methods, the phase data are used to detect susceptibility differences between tissues and are combined to the magnitude data to improve image contrast (Haacke et al., 2004). Increased iron accumulation in PD patients has been measured in deep gray nuclei using SWI (Zhang et al., 2009, 2010; Jin et al., 2011; Wu et al., 2014; Guan et al., 2016; Hopes et al., 2016). QSM is a more recent technique that converts the phase shifts to localize magnetic susceptibility (Haacke et al., 2015). Very little literature is available in PD rodent models applications; nevertheless, increased QSM has been reported in the SN of an MPTP mouse model (Guan and Feng, 2018). This study also evidenced that QSM was a more accurate method than *R*_2_^∗^ to detect iron-related changes in the SN, which was supported by a study including PD patients (Hopes et al., 2016). Further improvements of QSM methods have been developed for imaging the mouse brain microstructure at a very high resolution, such that striatal tracts can be reconstructed at 20-μm resolution based on QSM images from postmortem brains (Wei et al., 2016). It has therefore the potential to be used in PD models applications.

## MR Spectroscopy for Metabolism Evaluation

Magnetic resonance spectroscopy is based on the chemical shift and the spin–spin coupling effects. Different nuclei possess different resonant frequencies depending on their chemical environment and local magnetic fields. Their chemical shift is expressed in parts per million (ppm) relative to the standard reference compound tetramethylsilane (Barker et al., 2010).

Magnetic resonance spectroscopy has been used to assess brain metabolic changes in PD models. For example, increased GABA levels have been measured in the STR of MPTP mice and of 6-OHDA rats injected in the MFB (Chassain et al., 2010; Coune et al., 2013). Those results were consistent with human data in which increased GABA levels were found in the pons, putamen, and in the SN of patients (Emir et al., 2012; Gröger et al., 2014). Those findings could be explained by the following mechanism: the STR receives DA projections from the SNc, and knowing that DA inhibits GABAergic spiny neurons via D_2_ receptors in the STR, DA denervation should lead to hyperactivation of those neurons (Gerfen, 1992).

Likewise, Glu and glutamine (Gln) levels were found to increase in the STR of MPTP mice (Chassain et al., 2010), consistent with increased Glu in the SN of PD patients (Gröger et al., 2014). In their article, Chassain et al. (2010) explain that this Glu increase inducing changes in the corticostriatal activity is “related to an increased synthesis and release of Glu in the synaptic terminal of the STR.” In contrast, decreased Glu levels were measured in the STR of 6-OHDA rats injected in the MFB (Coune et al., 2013).

Decreased NAA levels were found in the ipsilateral STR and in the cortex of 6-OHDA rats injected in the MFB and SN, respectively (Hou et al., 2010; Coune et al., 2013), consistent with neuronal loss also reported in PD patients (Levin et al., 2012; Graff-Radford et al., 2014). However, no changes in NAA levels were found in an MPTP mouse model (Chassain et al., 2010).

Viral vector-based α-synuclein rodent models can be used as an alternative to transgenic models to produce α-synuclein accumulation and subsequent DA cell loss. Intranigral injections of an adeno-associated viral vector coding for human α-synuclein resulted in increased GABA levels in the STR as measured by MRS in rats (Coune et al., 2013). This finding was also reported in a 6-OHDA rat model by the same group and was consistent with MRS data in patients (Emir et al., 2012; Gröger et al., 2014). Furthermore, decreased NAA levels were measured in the SN of rats following intranigral injections. This finding was consistent with nigral cell loss induced in the model and with NAA decrease also found in patients (Clarke and Lowry, 2001; Öz et al., 2006).

Metabolic changes have also been observed in *PINK1* knockout rats using MRS. Villeneuve et al. (2016) found decreased taurine and Cre in the STR of *PINK1* rats. Similarly, decreased taurine and increased Gln were reported in the STR in the same model by Ren et al. (2019). Decreased taurine has been found in patients (Engelborghs et al., 2003) and is associated with mitochondrial function (Hansen et al., 2010). Increased Gln could be attributed to Glu dysregulation as found in the STR of patients (Gardoni and Bellone, 2015).

## Suggested Future Directions

### Evaluation of Neuroinflammation

Inflammation is common to many brain diseases as it has been shown to contribute to neurodegeneration (Ransohoff, 2016). To improve our understanding of the role of inflammation in the etiology of PD, lipopolysaccharide (LPS) animal models have been developed.

#### LPS Animal Model

The endotoxin LPS is a large molecule found in the outer membrane of Gram-negative bacteria. It binds the CD14/TLR4/MD2 (cluster of differentiation 14/Toll-like receptor 4/myeloid differentiation factor 2) receptor complex, which triggers the activation of proinflammatory pathways and ultimately the activation of microglia (Park and Lee, 2013).

Lipopolysaccharide can be injected in the central or peripheral nervous system to generate various inflammatory responses associated with neurodegeneration (see review by Batista et al., 2019). To induce Parkinson-like features, LPS is injected in the SN or STR, leading to DA degeneration and motor dysfunction in rats (Castano et al., 1998), as well as to macrophage and microglia reactions (Herrera et al., 2000; Gao et al., 2002). Likewise, systemic LPS injections in mice cause microglial activation, progressive loss of nigral DA neurons, and locomotor deficits (Qin et al., 2007).

Lipopolysaccharide models can also be used to assess mitochondrial dysfunction and their contribution to PD pathophysiology. For example, intrastriatal injections of LPS lead to energy dysfunction and neuronal loss in the STR (Hunter et al., 2017). Furthermore, LPS injections can change iron and ferritin levels in nigral glial cells of rats associated with decreased tyrosine hydroxylase staining in the GP and STR (Zhang et al., 2005; Hunter et al., 2008). Interestingly, transgenic mice overexpressing α-synuclein and injected with LPS show increased protein aggregation, chronic nigral DA neuronal loss, and nigral inflammation compared to wild-type mice, suggesting a potentiation role of inflammation on α-synuclein dysfunction (Gao et al., 2011).

In their review, Belloli et al. (2020) give an overview of neuroinflammation imaging markers in PD. Based on the work by Ostrerova-Golts et al. (2000), which provides further data that support the role of toxic iron in α-synuclein aggregation (Ostrerova-Golts et al., 2000), the authors suggest a link between iron concentration and neuroinflammation (Belloli et al., 2020). As SWI can measure iron accumulation, it can therefore be used to assess iron-driven neuroinflammation.

Overall, the use of animal models with LPS-induced inflammation can help our understanding of the neuroinflammatory component of PD. To date, no MRI studies have been conducted on LPS rodent models of PD, which opens new paths to the field. Combined MR methods have the potential to investigate both inflammation and neurodegeneration and to help better define the role of inflammation in the pathophysiology of PD.

#### Diffusion MR Spectroscopy of Metabolites

Among other complex mechanisms, inflammation involves glial cells activation, and it participates both to the clearance of damaged tissue and to tissue repair. Whether astrocytes play a protective role against inflammation or triggers it is still unknown and constitutes the topic of much ongoing research (Sofroniew, 2015). As brain metabolites are present in specific cell types, they can provide cell-specific markers. For instance, increased levels of choline (Cho) and mIns have been attributed to inflammation and gliosis, respectively (Öz et al., 2014). However, while metabolite concentration changes derived from conventional MRS cannot be attributed to specific pathological mechanisms, diffusion-weighted MRS can probe microstructural changes, such as glial cell swelling upon activation (Palombo et al., 2018). This emerging technique is based on the diffusion properties of metabolites within the intracellular space. For instance, metabolites in hypertrophic cells have more space to diffuse, which should increase their ADC—derived from multiple diffusion spectroscopy experiments.

The diffusivities of Cho and mIns have been proposed as specific markers of cellular hypertrophy triggered during glial activation (Ercan et al., 2016; Ligneul et al., 2019). Ligneul et al. (2019) used a cytokine ciliary neurotrophic factor-induced mouse model in which only hypertrophic reactive astrocytes were detected, whereas neuronal death and microglia were absent. They evidenced that the diffusivity of mIns was the most sensitive and specific marker of astrocytes morphological modulations in those mice.

### Evaluation of Neuromelanin

Neuromelanin is the intracellular pigmentation present in nigral DA neurons. While its accumulation in the SN during aging is normal, it is known that its deposition beyond a certain threshold and its specific degeneration can be a marker of the disease (Vila, 2019; Vila et al., 2019). Imaging methods sensitive to neuromelanin have been developed in humans including first spin echo T_1_-weighted MRI (Sasaki et al., 2006), and then magnetization transfer MRI after the magnetization transfer effect was found to be responsible for the neuromelanin contrast (Ogisu et al., 2013; Langley et al., 2015). The combination of multicontrast MRI such as SWI and magnetization transfer for iron (found in large amounts in the SNr) and neuromelanin (found in the SNc) detection, respectively, has been used to improve the delineation of the SN structures (Langley et al., 2015). Furthermore, diffusion-based tractography can add critical information on structural connections within the BG to better segregate the SNr from the SNc (Menke et al., 2010).

Multicontrast imaging can more easily be performed in anesthetized animals than in humans as scan time is less limited. In addition, the use of ultrahigh magnetic fields can drastically improve the visualization of the SN through increased signal-to-noise ratio (therefore resolution) and increased contrast, as reviewed in Lehéricy et al. (2014). It is therefore expected that the use of 11.7 T and 17 T scanners in rodents should be highly beneficial for better visualizing the details of the SN anatomy.

While neuromelanin is present in the human SNc, its absence in rodents (Marsden, 1961; Barden and Levine, 1983) prevents any investigation in those models. The group of M. Vila recently developed a rat model in which an adeno-associated viral vector expressing human tyrosinase is stereotaxically injected in the SNc region of their brains. Subsequently, those rats overexpressing human tyrosinase produce neuromelanin in the nigral DA neurons (Carballo-Carbajal et al., 2019). This model opens tremendous opportunities for the development of preclinical neuromelanin imaging strategies.

## Conclusion and Discussion

The changes in the MRI and MRS measures in the different rodent models presented are summarized in Table 1. The discrepancies found in the studies can be explained by several factors: the choice of a genetic or toxic model. Genetic models give insights into widespread cerebral alterations, but they lack the neurodegeneration component for most of them, limiting symptomatic evaluations. In toxic models, the injection site can produce different types of degeneration—massive or partial, rapid or progressive—and can have contrasting effects on the MR measures. The anesthesia protocols, isoflurane or medetomidine alone or a combination of both, which have different modes of action, can impact the rs-fMRI output measures (Williams et al., 2010; Schroeter et al., 2014). The detection sensitivity can also be modulated by different magnetic field strengths (from 7 to 11.7 T) and varying spatial resolutions used in those studies. The effect of iron on water diffusivity can also bias the measurements. Indeed, the presence of iron in the brain causes local magnetic field disturbances, which can lead to reduced water diffusivity if measured by imaging sequences sensitive to those local magnetic changes. In addition, it has been shown that the level of signal-to-noise ratio has an impact on anisotropy measurements, leading to overestimated high eigenvalues and underestimated low eigenvalues (Pierpaoli and Basser, 1996; Anderson, 2001). Therefore, one could expect to find higher FA in low-signal and iron-rich structures such as the SN. For example, Xu et al. (2015) showed that iron accumulation correlated with decreased MD and increased FA in the putamen of healthy adults. All of those considerations highlight the fact that the interpretation of diffusivity measures is complex and influenced by various mechanisms, such as iron accumulation in the SN and other regions involved in PD, which further contributes to the discrepancies found in the literature. Multimodal imaging combining susceptibility and diffusion sequences may help disentangle the relationship between iron and water diffusivity and could add valuable insights into human and rodent investigations. For instance, Du et al. (2011) demonstrated improved sensitivity and specificity of combined *R*_2_^∗^ and FA measures over the use of single measures to differentiate PD patients from healthy controls.

**TABLE 1:** Summary of the MRI/MRS measure changes with the corresponding rodent models.

	Method	Measure	Alterations	Model
Neurodegeneration	Diffusion	FA	Decreased in ipsilateral SN^1^	6-OHDA in MFB
			Decreased in bilateral SN^2^	6-OHDA in MFB
			Decreased in basal ganglia and all regions^3^	Tg PINK1
			Decreased in SN and CC^4^	Tg MitoPark
			Increased in ipsilateral SN^5^	6-OHDA in STR
			Increased in bilateral STR^6^	6-OHDA in STR
		MD	Increased in ipsilateral SN^1^	6-OHDA in MFB
			Increased in bilateral SN^2^	6-OHDA in MFB
			Increased in ipsilateral STR^6^	6-OHDA in STR
			Decreased in SN, STR, SM, TH^7^	Tg α-synuclein
		AD	Decreased in bilateral SN^2^	6-OHDA in MFB
			Decreased in basal ganglia and more regions^3^	Tg PINK1
			Decreased in SN, STR, SmCx, TH^7^	Tg α-synuclein
		RD	Increased in bilateral cortex^2^	6-OHDA in MFB
			Decreased in basal ganglia and more regions^3^	Tg PINK1
			Decreased in STR, Hc, TH^7^	Tg α-synuclein
	rs-fMRI	FC	Decreased in interhemispheric STR^1^	6-OHDA in MFB
			Decreased in ipsilateral cortices^8^	6-OHDA in MFB
			Decreased between ipsilateral M1/contralateral TH^6^	6-OHDA in STR
			Decreased between TH and STR^3^	Tg PINK1
			Decreased corticocortical and striatocortical connections^9^	6-OHDA in STR
			Increased between ipsilateral STR/bilateral SM^1^	6-OHDA in MFB
			Increased in bilateral TH^8^	6-OHDA in MFB
			Increased between ipsilateral STR/GP, contralateral M1/GP, interhemispheric STR/GP^6^	6-OHDA in STR
			Increased between cerebellar nuclei^3^	Tg PINK1
Iron	T_2_*	Signal intensity T_2_*	Decreased in ipsilateral SN^10^	6-OHDA in MFB
			Decreased in ipsilateral STR^11^	6-OHDA in STR
			Decreased in SN, STR^4^	Tg MitoPark
	QSM	QSM	Increased QSM in SN^12^	MPTP
Metabolism	MRS	GABA	Increased in ipsilateral STR^13^	MPTP
			Increased in ipsilateral STR^14^	6-OHDA in MFB
			Increased in ipsilateral STR^15^	AAV α-synuclein
		Glu	Increased in ipsilateral STR^13^	MPTP
			Decreased in ipsilateral STR^13^	6-OHDA in MFB
		Gln	Increased in STR^13^	MPTP
			Increased in STR^16^	Tg PINK1
		NAA	Decreased in ipsilateral STR^14^	6-OHDA in MFB
			Decreased in cortex^15^	6-OHDA in SN
			No change^13^	MPTP

*Rs-fMRI, resting-state functional MRI; MRS, magnetic resonance spectroscopy; FA, fractional anisotropy; MD, mean diffusivity; AD, axial diffusivity; RD, radial diffusivity; FC, functional connectivity; GABA, γ-aminobutyric acid; Glu, glutamate; Gln, glutamine; SN, substantia nigra; STR, striatum; M1, primary motor cortex; TH, thalamus; GP, globus pallidus; MFB, medial forebrain bundle; SM, sensorimotor cortex; Hc, hippocampus; CC, corpus callosum; Tg, transgenic; AAV, adenoviral vector. ^1^Monnot et al., 2017; ^2^Soria et al., 2011; ^3^Cai et al., 2019; ^4^Cong et al., 2016; ^5^Van Camp et al., 2009; ^6^Perlbarg et al., 2018; ^7^Khairnar et al., 2015; ^8^Westphal et al., 2017; ^9^Zhurakovskaya et al., 2019; ^10^Olmedo et al., 2017; ^11^Virel et al., 2014; ^12^Guan and Feng, 2018; ^13^Chassain et al., 2010; ^14^Coune et al., 2013; ^15^Hou et al., 2010; ^16^Ren et al., 2019.*

**FIGURE 4:**
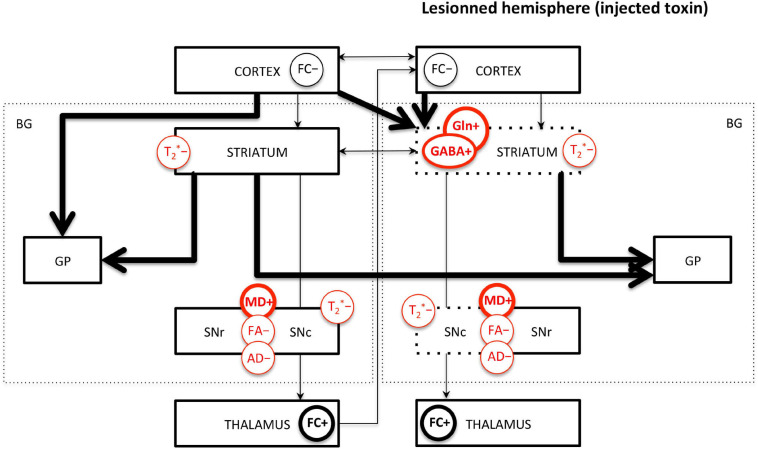
Main MRI/MRS findings in PD rodents affecting the basal ganglia pathways. FC, functional connectivity; MD, mean diffusivity; FA, fractional anisotropy; AD, axial diffusivity; GABA, γ-aminobutyric acid; Gln, glutamine; T_2_*, effective transverse relaxation time. “+” means increase and “-” means decrease of the MR measure. Measures indicated in red are commonly found across studies. Thick/thin lines represent increased/decreased measure. Arrows represent FC changes between structures. For toxic models, the dotted lines represent the lesioned structures; the left/right side of the figure represents the intact/lesioned hemispheres.

The limited number of MRI studies in genetic models makes the comparison with toxic models difficult. However, it can be highlighted that decreased FA and AD in the SN and increased GABA and Gln in the STR seem to be the most robust measures across toxic and genetic models. Increased MD seems to be measured only in 6-OHDA models, not in genetic models; however, more studies are needed to confirm this trend. Interestingly, decreased FC and increased FC in various brain regions, as well as interhemispheric changes, are common findings in both toxic and genetic models and suggest overall functional reorganizations. Decreased T_2_^∗^ signal or value in the SN and STR is also common across studies. Those major findings are illustrated in Figure 4.

All of those imaging measures provide insight into the physiopathology of PD; however, the animal models used do not replicate the entire complexity of the disease. The results should therefore be interpreted with this knowledge. Toxin-based models have been the most widely used in rodents, especially the 6-OHDA rat model; however, genetic-based models are being increasingly used in neuroimaging studies, especially the α-synuclein mouse and the *PINK1* rat models. Gathering more MRI data in various genetic models will help improve our understanding of the role of pathogenic genes in PD. The ideal model should be progressive and age-dependent and include DA depletion together with motor dysfunction as the ones observed in PD patients, as well as inflammation, which is not the case in most models so far. As a consequence, improving animal models is sought after by different groups for either genetic-based rat models (Creed and Goldberg, 2018) or humanized rat models expressing neuromelanin (Carballo-Carbajal et al., 2019).

Ongoing work from various groups aims at improving the specificity of MR-based methods (Petiet et al., 2019). As described above, the diffusivity of mIns as a specific marker of astrogliosis triggers increasing interest for diffusion-weighted MRS developments. Furthermore, this technique has the potential to quantitatively evaluate cell sizes, fiber lengths, and diameters (of either neuronal or glial cells). Indeed, the use of different diffusion time scales allows the quantification of the different parameters governing molecular displacement. Measuring the ADC in the limit of ultrashort diffusion times (<1 ms) allows probing short-range restrictions and cytosol viscosity (Marchadour et al., 2012), whereas measuring ADC in the limit of long diffusion times allows probing long-range restrictions such as cell walls, such that cell geometry and size can be inferred (Najac et al., 2014; Valette et al., 2018). Those technical developments open new fields of investigations in MR-based methods, and they should help better understand the underlying mechanisms.

## Author Contributions

AP solely contributed to this review, performed bibliographic searches, elaborated the figures and table and wrote the manuscript.

## Conflict of Interest

The author declares that the research was conducted in the absence of any commercial or financial relationships that could be construed as a potential conflict of interest.

## Abbreviations

PD: Parkinson disease
MRI: magnetic resonance imaging
DA: dopamine
LRRK2: leucin-rich repeat kinase 2
SNCA: α-synuclein coding gene
PARK2: Parkin 2
PINK1: phosphatase and tensin (Pten)-induced kinase 1
rs-fMRI: resting-state functional MRI
FC: functional connectivity
*R*_2_^∗^: transverse relaxation rate (1/T_2_^∗^)
T_2_^∗^: transverse relaxation time
MRS: magnetic resonance spectroscopy
6-OHDA: 6-hydroxydopamine
MPTP: 1-methyl-4-phenyl-1,2,3,6-tetrahydropyridine
BBB: blood–brain barrier
fMRI: functional MRI
BOLD: blood oxygenation level-dependent signal
SWI: susceptibility-weighted imaging
QSM: quantitative susceptibility mapping
LPS: lipopolysaccharide
Tg: transgenic
Glu: glutamate
GABA: γ-aminobutyric acid
NAA: *N*-acetyl -aspartate
mIns: myo-inositol
Cre: creatine
Gln: glutamine
Cho: choline
MD: mean diffusivity
FA: fractional anisotropy
AD: axial diffusivity
RD: radial diffusivity
ADC: apparent diffusion coefficient
SNc: substantia nigra pars compacta
STR: striatum
GP: globus pallidus
SNr: substantia nigra pars reticulata
TH: thalamus
STN: subthalamic nucleus
MFB: medial forebrain bundle
M1: primary motor cortex
CC: corpus callosum
SM: sensorimotor cortex
Hc: hippocampus
GPi: globus pallidus interna
GPe: globus pallidus externa
BG: basal ganglia
cp: cerebral peduncle.
